# The Rise, Progress and Present Status of Dentistry

**Published:** 1867-12

**Authors:** H. F. Bishop

**Affiliations:** Worcester, Mass.


### ARTICLE II.

### *The Rise, Progress and Present Status of Dentistry.*

By H. F. Bishop, D.D.S., of Worcester, Mass.

(Continued.)

Two generations of the 19th century have now already passed away ; in the first thirty-three years we find comparatively slow progress made in dentistry ; France in this period had well qualified dentists and writers for the times, among whom were Delabarre, Desirabode, Maury, Laforgue, Duval and many more. London had her Fox, Murphy, Snell and others ; In this country Philadelphia had her Gardette, Fitch and the talented Hudson, the friend and companion of Tom Moore—a noble Irishman skillful and thorough in his work ; the first as is now supposed to fill the fangs of teeth to the apex. Baltimore had her Hayden the first President of the first dental college. Boston had her Greenwood, Flagg, Randall and Solomon Keep. Many others might be mentioned in the principal cities of the Union but previous to 1833, it is doubtful if our cities were as well supplied with capable dentists as were the large cities of England and France. But in reviewing the last thirty-three years coming up to the present time, we find that our country has taken the lead and maintains a representative of the profession in almost every large city of foreign countries. Indeed so much is due to Americans in the recent and rapid developement of modern dentistry that we are warranted in the statement that if there be par excellence an American speciality, it is dentistry.

This era is so familiar to gentlemen of the profession that little will be said of it except to speak of the features which have given it its impulses. In the year eighteen hundred and thirty-nine the late Prof. Chapin A. Harris of Baltimore petitioned the Legislature of Maryland to obtain a charter for a dental college, which was granted, and it has been in successful operation since.

Dentistry owes more to Prof. Harris than to any one man; his endeavors, lifted the calling from a mere mechanical art, and advanced it far on the highway of a liberal profession.

He himself was educated in the profession of medicine and at one time in successful practice, but his interest in dentistry led him to devote his energies exclusively to its advancement. His services were required in the family of General Jackson who had great respect for his professional ability.

That his biography is still unwritten is a cause for regret and shame.

Other dental colleges soon follow with commendable rivalry; we could not expect the late Prof. Townsend of Philadelphia to exist far away from a central point of interest in the science, so through him and others Philadelphia soon established a college. She has timber for two, and in a short time two are established, till at length the country has six of these institutions. I had the pleasure this spring of attending the commencements of four of them and saw about one hundred men graduated.

In the year eighteen hundred and forty the American Society of Dental Surgeons was formed in New York, it was a pioneer in the way of societies—had its stormy life and came to a close, not without its uses and benefits, for, from its ashes rose the American Convention in 1855, which flourished for a while, but in a few years lost its best impulses, and finally after the withdrawal of many talented members to aid in the formation of another society, the convention has at length, become located in the city of New York. In the year eighteen hundred and fifty-nine the delegate Association was formed mainly through the energetic action and perseverance of an influential Philadelphia Professor. It has drawn together the best talent in the country, and encourages local societies till we have them organized all over the land.

Another feature to be mentioned is our Journalism. In periodicals the American Journal of Dental Science, Recorder and News Letter, of the past, and the Register, Cosmos, Times, and American Journal of Dental Science lately revived, of the present, have done much for the present status of dentistry, and are wielding a powerful influence for its advancement. The British Journal of Dental Science, the Dental Review of London, and L'Art Dentaire of Paris may also be mentioned as influential journals.

Taking a review of the past year, we find among the developements, some of importance. Some interesting facts are continually being brought to our notice in Histology by microscopical research. Conservative dentistry has become more and more appreciated—a large per cent. of teeth hitherto extracted on account of alveolar abscess or extensive decay are now saved by medication and successful treatment. The wonderful power and capacity of periostial tissue in restoring and rebuilding ossific matter is just now attracting the attention of surgeons as well as dentists, both in this country and Europe. It is imperfectly understood as yet, but it is a most interesting study, which cannot fail to richly repay the earnest seeker after light and truth. A Histologian in our profession who writes on the subject with great enthusiasm, considers that osteoplastic cells form bone: and that if the power to reproduce bone resides in periosteum it must be in its special ability to produce osteoplasts, which are the histological elements out of which bone is produced.

The subject of anaesthesia has been better understood, while the application of local anaesthetics is eliciting much attention, and when fully tested, it is thought by some may become a valuable aid to our profession.

The several colleges have united their efforts to ensure a common basis of requirements for their graduates, which will elevate the standard of all, and should lead to great harmony and concert of action. They are for making their diplomas a necessity to the practitioner—and the profession ought to demand it as a safe-guard to their ranks as well as to the public. A new feature has been introduced by some, or one at least, which permits candidates to graduate after being examined, who have been in practice since the year 1852. It would be a mistake perhaps not to encourage it, but it is certainly a measure which is very liable to abuse, and thus to work injustice to the regular graduates.

It has already led to a division by the withdrawal of the Pennsylvania college from the association, that body having adopted resolutions which her representatives considered as a rebuke to their past course. These divisions which we naturally regret, are not always unprofitable however: if the spirit of competition and rivalry will stimulate to greater efforts in elevating the general requirements of these institutions perhaps we ought to be reconciled to schism.

It is not the diploma simply, but the scientific attainments which should be the aim of us all—which the appreciative public more and more demands, and which alone can give us the prestige we desire. The Missouri college which started last Fall had twenty-five matriculants before its first session closed, and when it shall elect two more professors it will be in full membership with the Association of Colleges. The check which has been put upon students by an exaction of two years pupilage, and other requirements, is a step in the right direction.

Prominent among the occurrences of the past year are the Legislative bills by the States of Ohio, North Carolina, Kentucky and Indiana, for the suppression of quackery in dentistry—it is to be hoped that these bills will become laws and be enforced. The Rubber question seems stretchy, and a recoil of its over-stretched capacity is expected to injure some of its most interested parties. The law of humanity demands an unrestricted use of anything so beneficial to mankind as rubber in supplying artificial teeth and in making interdental splints for fractured jaws. We agree with Dr. Evans in his letter from Paris three years ago to this association, that "professional science should never tolerate a patent within its domains." And, without doubt, this one will be universally and steadily resisted. Certainly no one can gainsay the sworn affidavits which he has recently sent, on which settles the priority of introduction. The rubber license contract which to say the least is wholly an exparte contract drawn entirely in the interests of the patentee, ignoring all rights of the dentists, and subjecting them to a degrading inquisition, and perpetual annoyance, is intolerable—even if all would be bound by it, but by its unequal action comes still harder on the few who feel compelled to pay its claims.

We rejoice that attention is being called to the importance of preserving the health of the dentist, for certainly in no profession are greater risks encountered. A prize of $200 recently offered for the best essay upon the subject, will doubtless bring forth many interesting and useful considerations. During the past year dentists have been regularly appointed on the staff of one or two of the Medical Hospitals in New York.

Gentlemen! But a short time since we were clamoring for recognition and equal footing with the liberal professions. To-day, I congratulate you upon having secured all we deserve, as co-laborers in a special science from those professions, and far more I fear than we can maintain properly with the meagre educational attainments that exist at present in our ranks. We have in contemplation a liberal and flattering arrangement for a college of dentistry in Massachusetts connected with Harvard College, which if we can inaugurate successfully by able men for Professors, will supply what has long been needed in this section of the country. Massachusetts ought to lead the way, and hold a supremacy in dentistry as in other sciences,—it remains to be seen whether we have the ability as her sons, to keep her fair name in the front rank.

A talented member of the profession recently asserted, that in the past six years dentistry had been completely revolutionized. This is true to a great extent;—one hardly knows which is the most difficult for an old practitioner to unlearn the old practice, or adopt the new in this age of progress. In the rush of new ideas, new methods, and new substances, we are tempted perhaps too much to experiments which may not be so fortunate for our patients, or, for our reputation.

We live in an era when nearly every department of manufactures and mechanism is characterized by poor and cheap work, which all springs from a neglect of the great moral obligation resting on every man to produce his best.

No one has a right to make a poor thing even for himself: it is a bad example, the evil effects of which are not confined to him alone. There is no mediocrity in God's work, and we are instructed to take Him as our great pattern.

Let us individually set ourselves against this tendency of the times and conscientionsly give our best efforts in every undertaking,

The pen though little used in modern dentistry as our meagre literature proclaims, seems an attractive instrument for the aspiring student; never was there a better opening for young men of talent and education, than in this age to acquire reputation in the profession. The great misfortune of many now in practice is a deficiency in an early, and thorough education to start with. The coming age will much improve in this direction, with the greater privileges, and facilities enjoyed, and the demand for, and compensation offered to, talented labor.

Our hands should not only be educated and our hearts interested in our work, but our minds should be active, and growing.

Said Webster the statesman: "If we work upon marble it will perish—if we work upon brass time will efface it; if we rear temples, they will crumble into dust; but if we work upon our immortal minds—if we endue them with principles—with the just fear of God, and our fellow men—we engrave on these tablets something which will brighten to all eternity."

